# Supplementary material for: Disentangling metabolic functions of bacteria in the honey bee gut
Source: PLoS Biol. 2017 Dec 12;15(12):e2003467. doi: 10.1371/journal.pbio.2003467 (PMC5726620; doi:10.1371/journal.pbio.2003467)
Supplement: S8 Data — (ZIP) [file pbio.2003467.s008.zip › Z-score_plots/999 microbial substrate Quercetin 3-O-beta-D-glucosyl-(1-2)-beta-D-glucoside.pdf]

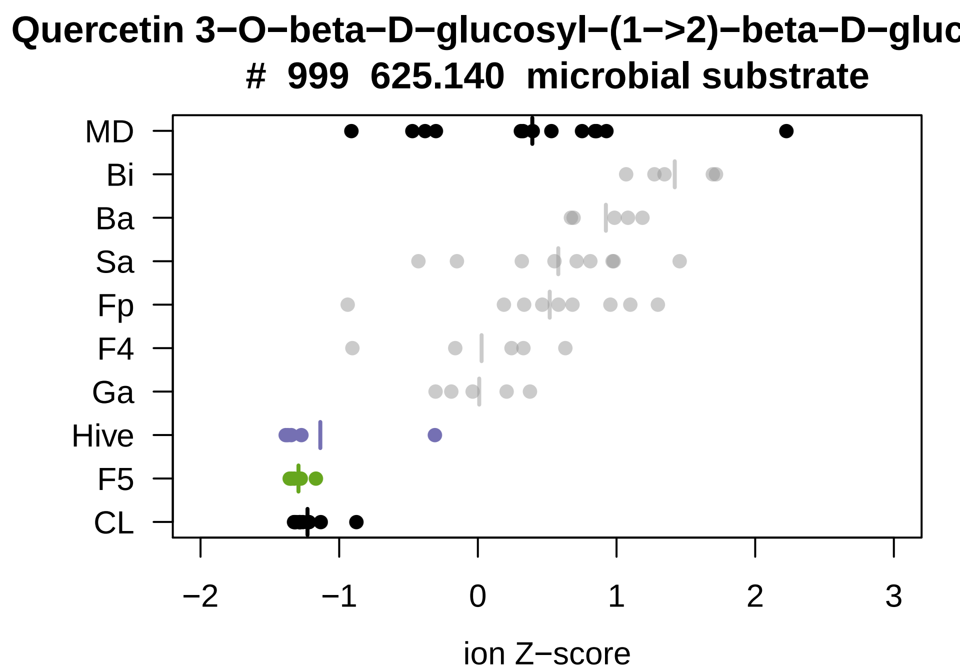
Quercetin 3−O−beta−D−glucosyl−(1−>2)−beta−D−gluc
999 625.140 microbial substrate
MD
Bi
Ba
Sa
Fp
F4
Ga
Hive
F5
CL
−2
−1
0
1
2
3
ion Z−score
